# *Drosophila simulans*: A Species with Improved Resolution in Evolve and Resequence Studies

**DOI:** 10.1534/g3.117.043349

**Published:** 2017-05-24

**Authors:** Neda Barghi, Raymond Tobler, Viola Nolte, Christian Schlötterer

**Affiliations:** * Institut für Populationsgenetik, Vetmeduni Vienna, 1210, Austria; † Vienna Graduate School of Population Genetics, Vetmeduni Vienna, 1210, Austria

**Keywords:** experimental evolution, evolve and resequence, *Drosophila simulans*, *Drosophila melanogaster*, chromosomal inversions

## Abstract

The combination of experimental evolution with high-throughput sequencing of pooled individuals—*i.e.*, evolve and resequence (E&R)—is a powerful approach to study adaptation from standing genetic variation under controlled, replicated conditions. Nevertheless, E&R studies in *Drosophila melanogaster* have frequently resulted in inordinate numbers of candidate SNPs, particularly for complex traits. Here, we contrast the genomic signature of adaptation following ∼60 generations in a novel hot environment for *D. melanogaster* and *D. simulans*. For *D. simulans*, the regions carrying putatively selected loci were far more distinct, and thus harbored fewer false positives, than those in *D. melanogaster*. We propose that species without segregating inversions and higher recombination rates, such as *D. simulans*, are better suited for E&R studies that aim to characterize the genetic variants underlying the adaptive response.

Standing genetic variation in natural populations underlies their potential to adapt to novel environments. The evolve and resequence (E&R) approach (Turner *et al.* 2011), which combines experimental evolution with sequencing of pooled individuals (Pool-Seq) (Schlötterer *et al.* 2014), provides an excellent opportunity to understand how this standing genetic variation is being used to fuel adaptation of the evolving populations (Schlötterer *et al.* 2015; Long *et al.* 2015). Because experimental evolution permits the analysis of replicate populations, which have evolved from the same standing genetic variation under identical culture conditions, it is possible to distinguish selection from random, nondirectional changes (Kawecki *et al.* 2012; Schlötterer *et al.* 2015).

A short generation time and high levels of polymorphism, in combination with a small, well-annotated genome, has made *Drosophila* *melanogaster* the preferred sexual model organism to study the genomic response to truncating selection. Many traits such as aging (Remolina *et al.* 2012), courtship song (Turner *et al.* 2013), hypoxia (Zhou *et al.* 2011; Jha *et al.* 2016), body size (Turner *et al.* 2011), egg size (Jha *et al.* 2015), development time (Burke *et al.* 2010; Graves *et al.* 2017), and *Drosophila* C virus (DCV) resistance (Martins *et al.* 2014) have already been studied. The E&R approach has also been applied to laboratory natural selection experiments, in which differential reproductive success is the sole driver of adaptation to novel environments such as elevated temperature (Orozco-terWengel *et al.* 2012; Tobler *et al.* 2014; Franssen *et al.* 2015) and high cadmium and salt concentration (Huang *et al.* 2014). For traits with a simple genetic basis, such as DCV resistance, E&R has identified causal genes (Martins *et al.* 2014); on the other hand, identification of the genetic basis of polygenic traits has been considerably more challenging because of the large size of the genomic regions that have been identified (Burke *et al.* 2010; Turner *et al.* 2011; Zhou *et al.* 2011; Orozco-terWengel *et al.* 2012; Remolina *et al.* 2012; Tobler *et al.*, 2014). These genomic regions often contain a substantial number of candidate SNPs that are mostly false positives (Nuzhdin and Turner 2013; Tobler *et al.* 2014; Franssen *et al.* 2015). The inflated numbers of false positives can be partly attributed to linkage disequilibrium (LD) and long-range hitchhiking caused by low frequency adaptive alleles (Tobler *et al.* 2014; Franssen *et al.* 2015). Other important factors contributing to the large number of false positives include (1) reduced recombination rates close to the centromeres, and (2) the presence of large chromosomal inversions that suppress recombination and occasionally also respond to selection (Kapun *et al.* 2014).

*D. simulans*, a sister species of *D. melanogaster*, lacks large segregating inversions (Aulard *et al.* 2004), has higher recombination rate, and the centromeric recombinational suppression is restricted to a much smaller part of the chromosomes (True *et al.* 1996). These characteristics make *D. simulans* potentially more suitable for E&R studies (Kofler and Schlötterer 2014; Tobler *et al.* 2014). While the availability of genomic and functional resources is not comparable to *D. melanogaster*, improved genome assemblies and annotations are available for *D. simulans* (Hu *et al.* 2013; Palmieri *et al.* 2015).

In this study we contrast the genomic response of a *D. simulans* E&R study spanning 60 generations to *D. melanogaster* populations that have been evolving for the same length of time in the same hot temperature environment. Consistent with the absence of segregating inversions and higher recombination rate, the selection signatures in *D. simulans* result in substantially smaller genomic regions carrying putatively selected variants.

## Materials and Methods

### D. simulans experimental populations and selection regimes

202 isofemale lines were established from a natural *D. simulans* population collected in Tallahassee, Florida in November 2010. The isofemale lines were maintained in the laboratory for nine generations prior to the establishment of the founder populations to rule out infections and determine the species. Five mated females from each isofemale line were used to establish 10 replicates of the founder population, three of which were used in our study. They were maintained as independent replicates with a census population size of 1000 and ∼50:50 sex ratio. Both temperature and light was cycled every 12 hr between 18 and 28°, corresponding to night and day.

### Genome sequencing, mapping, and SNP calling of D. simulans data

Genomic DNA was prepared for three founder replicates (females only) and three replicates of the evolved populations at generation 60 (mixed sexes). Details of DNA extraction and library preparation are summarized in Supplemental Material, Table S1. The average genome-wide sequence coverage across the founder and evolved population replicates was ∼259× and ∼100×, respectively.

Reads were trimmed using ReadTools version 0.2.1 (https://github.com/magicDGS/ReadTools) to remove low quality bases (Phred score <18) at 3′ end of reads (parameters: --minimum-length 50 --no-5p-trim --quality-threshold 18 --no-trim-quality). The trimmed reads were mapped using bwa (version 0.5.8c; aln algorithm; parameters: -o 1 -n 0.01 -l 200 -e 12 -d 12) (Li and Durbin 2009) to the *D. simulans* reference genome (Palmieri *et al.* 2015) on a Hadoop cluster with Distmap version 1.0 (Pandey and Schlötterer 2013). Reads in the bam files were sorted and duplicates were removed with Picard version 1.140 (http://broadinstitute.github.io/picard). Reads with low mapping quality and improper pairing were removed (parameters: -q 20 -f 0x0002 -F 0x0004 -F 0x0008) and the bam files were converted to mpileup files using SAMtools version 1.2 (Li *et al.* 2009). The mpileup files were converted to a synchronized pileup file using PoPoolation2 (parameter: --min-qual 20) (Kofler *et al.* 2011). Furthermore, repeats (identified by RepeatMasker, http://www.repeatmasker.org) and 5-bp regions flanking indels (identified by PoPoolation2: identify-genomic-indel-regions.pl --indel-window 5 --min-count 5) were masked using PoPoolation2 (identify-indel-regions.pl --min-count 2% of the average coverage across all founder libraries).

SNPs were called from the founder populations; in brief, initially the SNPs with minimum base quality of 40 present in at least one replicate of the three founder populations were selected for further analyses. To improve the reliability of the pipeline, the polymorphic sites lying in the upper and lower 1% tails of the coverage distribution (*i.e.*, ≥423× and ≤11×, respectively; upper tail based on the library with the highest sequencing depth, lower tail estimated from total coverage of all replicates and time points) were removed. Further, we masked 200-bp flanking SNPs specific to autosomal genes translocated to the Y chromosome (R. Tobler, V. Nolte, and C. Schlötterer, unpublished data). In total, 4,391,296 SNPs on chromosomes 2 and 3 were used for subsequent analysis [644,423 SNPs on X chromosome were used for effective population size (*N_e_*) estimation but were excluded from other analyses, see below]. For all SNP sites remaining after the filtering steps, we determined the allele frequencies using only reads with a quality score of at least 20 at the SNP position.

### Genome sequencing and mapping of D. melanogaster data

The *D. melanogaster* data used in this study are part of an ongoing experiment (founder population from Orozco-terWengel *et al.* 2012; F59 populations from Franssen *et al.* 2015). Similar to *D. simulans*, temperature and light was cycled every 12 hr between 18 and 28°, corresponding to night and day. To increase the coverage of libraries for the founder populations, additional sequencing was performed (see Table S1 for details of DNA extraction and library preparation). The final average genome-wide coverage of the founder and evolved populations was ∼190× and ∼83×, respectively. Read processing and mapping are described in Tobler *et al.* (2014). Similar to the *D. simulans* data set, SNPs were called from the base population with base quality of 40. Then, SNPs lying in the upper and lower 1% tails of the coverage distribution (*i.e.*, ≥328× and ≤9×, respectively; upper tail based on the library with the highest sequencing depth, lower tail estimated separately from total coverage of all replicates and time points) were removed. 2,934,945 SNPs on chromosomes 2 and 3 were used for further analyses. SNPs on the X chromosome (408,982) were only used for *N_e_* estimation. Allele frequencies were determined based on reads with base quality of at least 20.

### Candidate SNP inference in D. simulans and D. melanogaster

To compare the selected genomic regions between *D. simulans* and *D. melanogaster*, the sequencing reads were downsampled using Picard (DownsampleSam, http://broadinstitute.github.io/picard) to obtain similar mean genome-wide coverage of the libraries in both species (Table S2). To identify SNPs with pronounced allele frequency changes (AFC), we contrasted the founder and evolved populations (at generation 60 for *D. simulans* and 59 for *D. melanogaster*) using the Cochran–Mantel–Haenszel (CMH) test (Agresti 2002). For each species, we estimated *N_e_* in windows of 1000 SNPs across all chromosomes and replicates using Nest (function estimateWndNe, method Np.planI; Jónás *et al.* 2016). Averaging the medians of the *N_e_* values across replicates, we obtained the *N_e_* estimate for autosomes and the X chromosome of each species. The estimated *N_e_* for the X chromosome (*N_e_* = 224) was approximately three-quarters of autosomes (*N_e_* = 285) in *D. simulans*, whereas in *D. melanogaster*, the *N_e_* of the X chromosome (*N_e_* = 301) was 1.5 times higher than that of the autosomes (*N_e_* = 201). This discrepancy in *D. melanogaster* has been noted before (Orozco-terWengel *et al.* 2012; Jónás *et al.* 2016) and has been attributed to an unbalanced sex ratio, background selection, and a larger number of SNPs being affected by selection on the autosomes. Because it is not clear whether these pronounced differences reflect differences in selection or mating patterns, we excluded the X chromosome from the analysis. Since the CMH test does not account for drift, we inferred candidate SNPs by simulating drift based on the inferred autosomal *N_e_* estimates and determined an empirical CMH cutoff using a 2% false positive rate. Forward Wright–Fisher simulations were performed with independent loci using Nest (function wf.traj; Jónás *et al.* 2016). The simulation parameters (*i.e.*, number of SNPs, allele frequencies in the founder populations, coverage of libraries, *N_e_*, and number of replicates and generations) matched the experimental data. To infer the genomic regions under selection, we computed the average *p*-value of all candidate SNPs (above the empirical CMH cutoff: 31 for *D. simulans* and 27.32 for *D. melanogaster*) in 200-kb sliding windows with 100-kb overlap. Adjacent windows with the average *p*-value above the CMH cutoff were merged.

### Data availability

The raw reads for all populations are available from the European Sequence Read Archive under the accession numbers mentioned in Table S1. SNP data sets in sync format (Kofler *et al.* 2011) are available from the Dryad Digital Repository under http://dx.doi.org/10.5061/dryad.p7c77.

## Results

**Figure 1:**
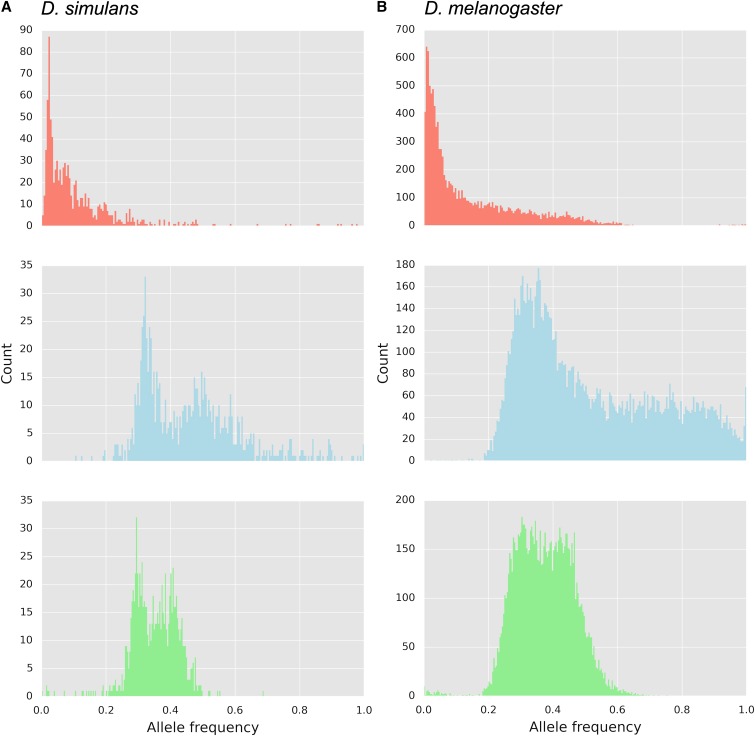
Allele frequency distribution of candidate SNPs averaged across replicates in (A) *D. simulans* and (B) *D. melanogaster*. Founder population (top panels), generation 60/59 (middle panels), and frequency change (bottom panels) of candidate SNPs. Candidate SNPs were determined from an empirical 2% false positive rate determined by neutral simulations assuming no linkage.

**Figure 2:**
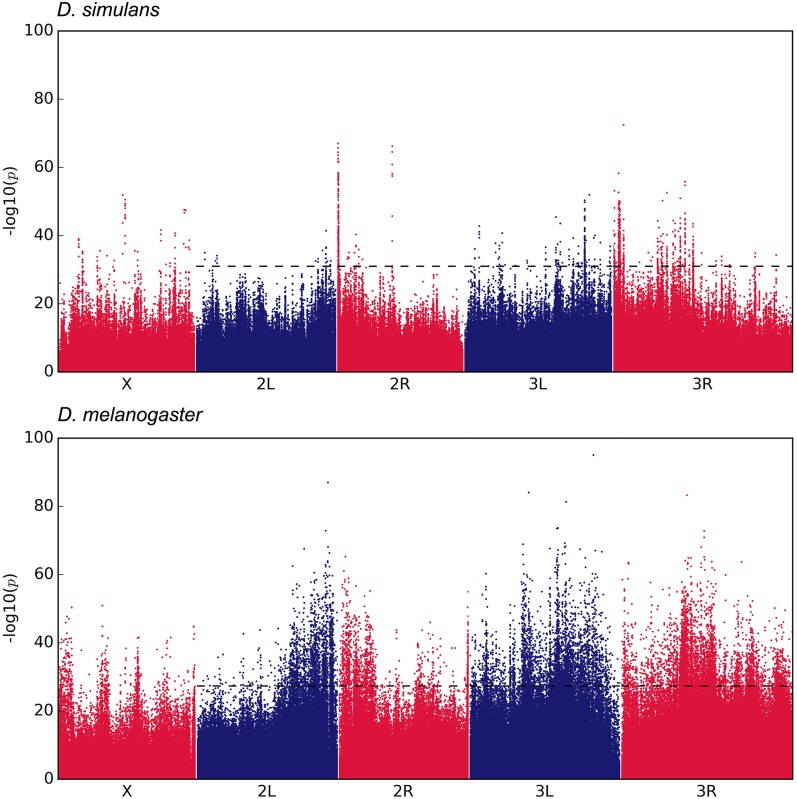
The genomic distribution of candidate SNPs in *D. simulans* (top panel) and *D. melanogaster* (bottom panel): The Manhattan plots show the negative log_10_-transformed *p*-values of SNPs corresponding to the genomic positions. The *p*-values were determined using CMH test by comparing the founder and evolved populations using the same sequencing coverage for both species. The dotted lines show the CMH cutoff based on empirical 2% false positive rate determined by neutral simulations assuming no linkage. Because the relative *N_e_* estimates of X chromosomes and autosomes were nonconcordant between both species, we did not determine outlier SNPs for the X chromosome.

Three replicates of a *D. simulans* founder population were maintained in a hot temperature environment for 60 nonoverlapping generations. We sequenced pooled individuals of the three founder populations and three evolved populations, and compared these data to *D. melanogaster*, which evolved for 59 generations under the identical selection regime (Orozco-terWengel *et al.* 2012; Franssen *et al.* 2015). SNPs with pronounced AFC across the three replicates were identified with the CMH test by contrasting the founder and evolved populations of each species. While the CMH test is a powerful tool for identifying putative targets of selection (Kofler and Schlötterer 2014), it is not sufficient for determining which outlier loci are deviating from neutral expectations. Consequently, we estimated the *N_e_* for each species based on genome-wide AFC between founder and evolved populations (Jónás *et al.* 2016). We then performed neutral simulations with the predicted *N_e_* for autosomes to derive an empirical CMH cutoff based on a 2% false positive rate. We identified 918 candidate SNPs in *D. simulans*; whereas in *D. melanogaster*, 11,115 SNPs were identified as outliers (Figure 1 and Figure 2). In both species, the majority of candidate SNPs start from a low frequency. *D. melanogaster* has more SNPs starting at intermediate frequencies that reach higher frequencies after 59 generations (Figure 1). Nonetheless, despite the rapid frequency change in response to the hot environment, only a small fraction of candidate SNPs (1.2% in *D. simulans* and 6.3% in *D. melanogaster*) approached fixation (major allele frequency ≥0.9) after 60 generations.

**Figure 3:**
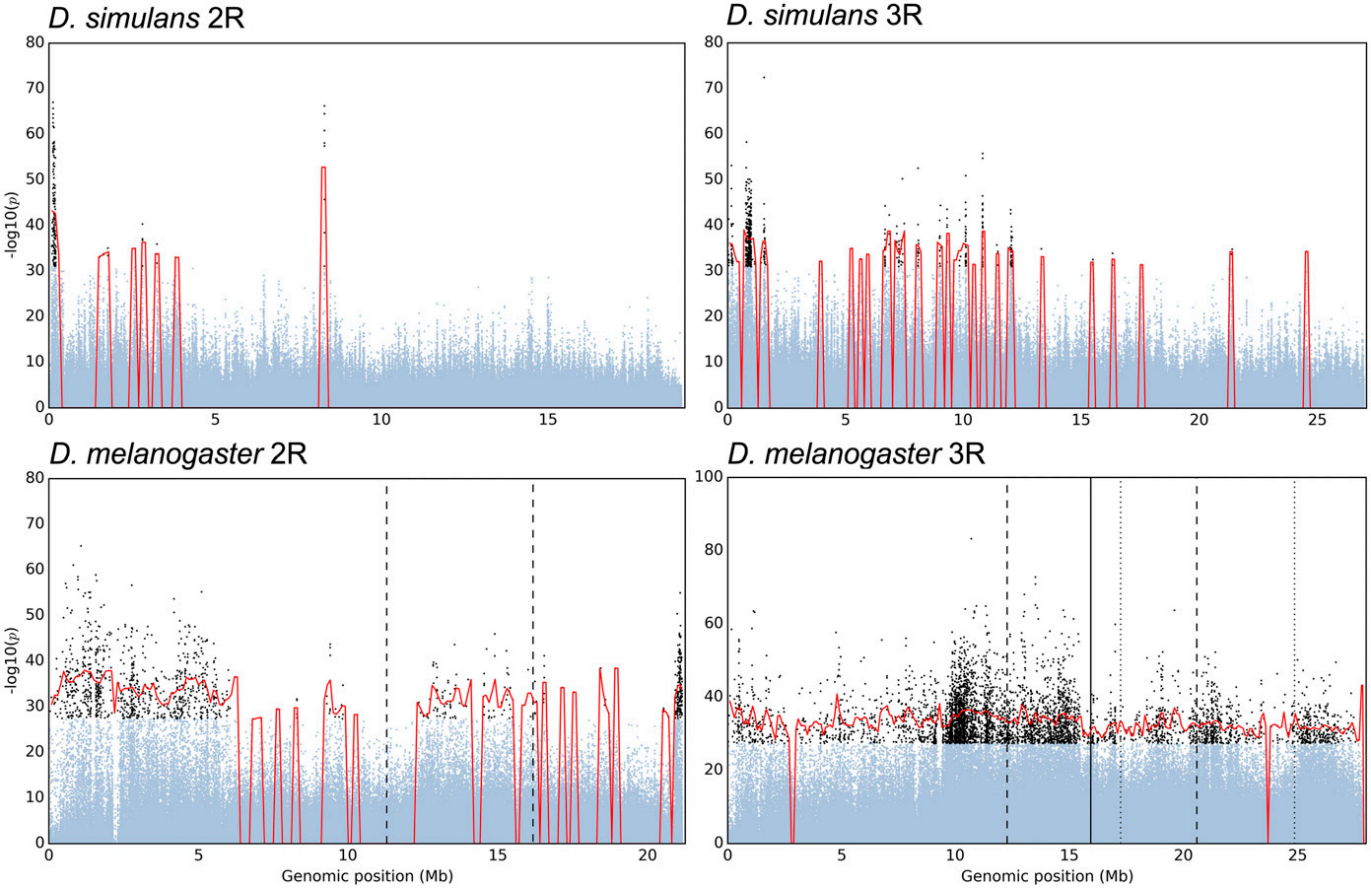
Identification of selected regions on two chromosome arms: Manhattan plots of chromosome arms 2R (left panels) and 3R (right panels) are shown for *D. simulans* (top panels) and *D. melanogaster* (bottom panels). The CMH *p*-values of candidate SNPs (black dots) were averaged across 200-kb windows, over sliding intervals every 100 kb. Adjacent windows with average *p*-values above CMH cutoffs (see *Materials and Methods*) were merged (red lines). Boundaries of the inversion in 2R [*In*(*2R*)*Ns*] are shown in dashed lines. Three overlapping inversions in 3R, *i.e.*, *In*(*3R*)*Payne*, *In*(*3R*)*Mo*, and *In*(*3R*)*C* are indicated with dashed, dotted, and solid lines, respectively.

Neutral SNPs linked to a target of selection change their frequencies more than expected by chance, which results in a characteristic peak structure observed in Manhattan plots. Such selection peaks can be recognized above the dotted line, which separates candidate SNPs in *D. simulans* based on the empirical 2% false positive rate from nonselected SNPs (Figure 2, upper panel). Visual inspection of the Manhattan plots for both species revealed that *D. simulans* had narrower and more distinct peak structures than *D. melanogaster* (Figure 2). While a pronounced peak structure narrows the genomic region affected by selection, a peak also indicates that SNP-based analyses are not informative and can be misleading: many nonselected SNPs show a selection signature due to linkage with the target of selection. Thus, the identification of peak structures is a prerequisite for determining selection targets. To this end, we explored several peak finding procedures (*e.g.*, Futschik *et al.* 2014; Beissinger *et al.* 2015), but complex selection signatures in our data sets makes this a challenging task, even for *D. simulans* which has much clearer peak structures. One of the challenges we encountered in our efforts to separate distinct peaks was that very narrow peaks were not recognized due to too few candidate SNPs. We therefore employed an approximate method to determine the fraction of the genome affected by selection. Averaging the *p*-value of all candidate SNPs in 200-kb sliding windows, we distinguished between regions influenced by directional selection from those evolving neutrally (Figure 3 and Figures S1 and S2 in File S1). Using this method, we detected 46 peaks covering ∼22.6 Mb (25.3% of chromosomes 2 and 3 of the reference genome) in *D. simulans*, and 31 peaks covering 84.4 Mb (87.4%) in *D. melanogaster*. Particularly striking is the difference between the two species on chromosome 3R, which contains three segregating, overlapping inversions [*In*(*3R*)*Payne*, *In*(*3R*)*Mo*, and *In*(*3R*)*C*] in the *D. melanogaster* population (Kapun *et al.* 2014). Almost the entire *D. melanogaster* 3R chromosome was characterized as a genomic region affected by selection, while in *D. simulans* several distinct peaks could be recognized (Figure 3). Moreover, in chromosome 2 of *D. melanogaster*, the regions near the centromere spanning to both chromosome arms contained numerous candidate SNPs forming broad peaks, probably due to a reduced recombination in this region (Figure 2, Figure 3, and Figure S1 in File S1).

## Discussion

The different genomic signatures of *D. simulans* and *D. melanogaster* induced by adaptation to high temperature can be attributed to species-specific characteristics and the design of the experimental study. Factors such as chromosomal inversions and low recombination regions can be associated with broad genomic regions determined to be under selection in *D. melanogaster*. Large chromosomal inversions are common in natural *D. melanogaster* populations, suppressing recombination over extensive genomic regions (Kirkpatrick 2010). In the *D. melanogaster* experimental populations, these inversions have contributed in two ways to the large number of observed candidate SNPs: first, the suppression of recombination has resulted in the association of SNPs effectively across entire chromosomes—even under the influence of drift alone. Second, inversion *In*(*3R*)*C* showed a consistent increase in frequency across multiple replicates, suggesting that it harbors, or is linked to, some selection targets (figure 4 in Kapun *et al.* 2014), exacerbating the impact of the inversions on chromosome 3R. On top of this, in *D. melanogaster* large parts of the chromosomes are affected by the reduced recombination rate toward the centromeres (True *et al.* 1996 and Comeron *et al.* 2012). Consistent with low recombination affecting the selection signature, we observed a broad peak near the centromere in chromosome 2 spanning both chromosome arms (Figure 2, bottom panel; Figure 3; and Figure S1 in File S1). The impact of recombination was also noted previously (Franssen *et al.* 2015), where low recombination regions were associated with high LD (1–10 Mb) in a *D. melanogaster* experimental evolution data set.

Because the selection signature in *D. melanogaster* extends to linked neutral SNPs over large genomic regions, almost no specific selected targets could be distinguished. However, several regions with presumably distinct selection targets could be identified for *D. simulans* (Figure 2 and Figure 3), a species that lacks large segregating inversions (Aulard *et al.* 2004) and has a 1.3× higher genome-wide recombination rate, with a much less pronounced recombination depression close to centromeres and telomeres (True *et al.* 1996). Contrasting the patterns of nucleotide polymorphism in natural populations of both species (Nolte *et al.* 2013), the difference in recombination landscape between *D. melanogaster* and *D. simulans* is evident (Figures S3–S6 in File S1), suggesting the smaller genomic region with suppressed recombination toward the centromeres in *D. simulans* contributes to a clearer selection signature.

In *D. melanogaster*, it has been proposed that LD and long-range hitchhiking caused by low frequency adaptive alleles result in a large number of false positive candidate SNPs (Tobler *et al.* 2014; Franssen *et al.* 2015). The *D. simulans* founder population had more LD than the *D. melanogaster* population (D. Gómez-Sánchez, R. Poupardin, V. Nolte, and C. Schlötterer, unpublished data; Figure S7 in File S1), which is most likely a consequence of their different demographic histories (Hamblin and Veuille 1999 and the references therein). This increased LD is expected to have the opposite effect, resulting in a higher mapping accuracy in *D. melanogaster*. However, our results indicate that despite higher LD in *D. simulans*, the genomic regions under selection in this species are still narrower than in *D. melanogaster*. One alternative explanation for the difference in mapping resolution of *D. melanogaster* and *D. simulans* may be that the genetic architecture of adaptation differs between the two species. Nevertheless, we consider this unlikely. First, most of the candidates in both *D. melanogaster* and *D. simulans* (Figure 1) start from low frequency, indicating that selection is acting on rare variants in both species. Hence, it is not likely that the selected alleles occurring at lower frequency in *D. melanogaster* would result in more hitchhiking of linked variants than in *D. simulans*. Second, in natural populations the genetic architecture seems to be similar between the two species. In North America and Australia, parallel clines have been described for *D. simulans* and *D. melanogaster* (Reinhardt *et al.* 2014; Zhao *et al.* 2015; Machado *et al.* 2016; Sedghifar *et al.* 2016). Remarkably, more genes share the pattern of clinal variation in both species than expected by chance (Reinhardt *et al.* 2014; Machado *et al.* 2016; Sedghifar *et al.* 2016). Furthermore, several clinal genes were also differentially expressed at high and low temperatures in both *D. melanogaster* and *D. simulans* (Zhao *et al.* 2015). While the strength and stability of the clines differ between *D. melanogaster* and *D. simulans*, the observation that both species share genes with clinal variation and differential expression in response to temperature treatment, strongly suggests that there are similarities in the genomic architecture of temperature adaptation between both species.

Computer simulations indicated that the mapping accuracy increases with the number of founder chromosomes (Baldwin-Brown *et al.* 2014; Kofler and Schlötterer 2014; Kessner and Novembre 2015). The founder population of *D. melanogaster* encompassed only 113 isofemale lines, while the *D. simulans* experiment was started from 202 isofemale lines. Notably, while it is difficult to determine to what extent the various factors have contributed to the higher resolution of the *D. simulans* E&R study, the presence of distinct peaks in *D. simulans* suggests that the large segregating chromosomal inversions, low recombination rate, and, likely, fewer founder chromosomes, among other factors, contributed most to the low resolution of the *D. melanogaster* E&R experiment.

While the higher recombination rate in *D. simulans* and the absence of segregating inversions support our observation that *D. simulans* may be better suited for E&R studies than *D. melanogaster*, it is important to keep in mind that we tested only a single selection regime, and for other traits *D. melanogaster* may have a cleaner selection response. While possible, we do not consider this very likely because other studies selecting for different traits in *D. melanogaster* also identified a large number of loci deviating from neutral expectations (Burke *et al.* 2010; Turner *et al.* 2011; Zhou *et al.* 2011; Orozco-terWengel *et al.* 2012; Remolina *et al.* 2012; Tobler *et al.* 2014).

Our results show that using inversion-free *D. simulans* with low recombination depression toward the centromeres improves the resolution of E&R studies, resulting in identification of narrower and more precise genomic regions under selection than in *D. melanogaster* (Orozco-terWengel *et al.* 2012; Tobler *et al.* 2014; Franssen *et al.* 2015). Even though the selection signatures in *D. simulans* were substantially more distinct than those in *D. melanogaster*, we caution that subsequent characterization of the selection targets is still challenging. More refined methods need to be developed that separate the selection signatures from adjacent targets of selection by accounting for the differences in starting frequencies of the SNPs and selection intensities. Thus, the comparison of selection targets between both species is not informative unless at least for one species the target of selection can be further narrowed down using, for example, expression profiling in combination with Pool-Seq selection signatures. Furthermore, improvements in the experimental design, *e.g.*, using more replicates and more founder chromosomes (Baldwin-Brown *et al.* 2014; Kofler and Schlötterer 2014; Kessner and Novembre 2015) can further increase the accuracy of mapping the selected targets.

## Supplementary Material

Supplemental material is available online at www.g3journal.org/lookup/suppl/doi:10.1534/g3.117.043349/-/DC1.
