# Supplementary material for: Drosophila simulans: A Species with Improved Resolution in Evolve and Resequence Studies
Source: G3 (Bethesda). 2017 May 24;7(7):2337–43. doi: 10.1534/g3.117.043349 (PMC5499140; doi:10.1534/g3.117.043349)
Supplement: Supplementary file 1 [file 2337FileS1.docx]

**
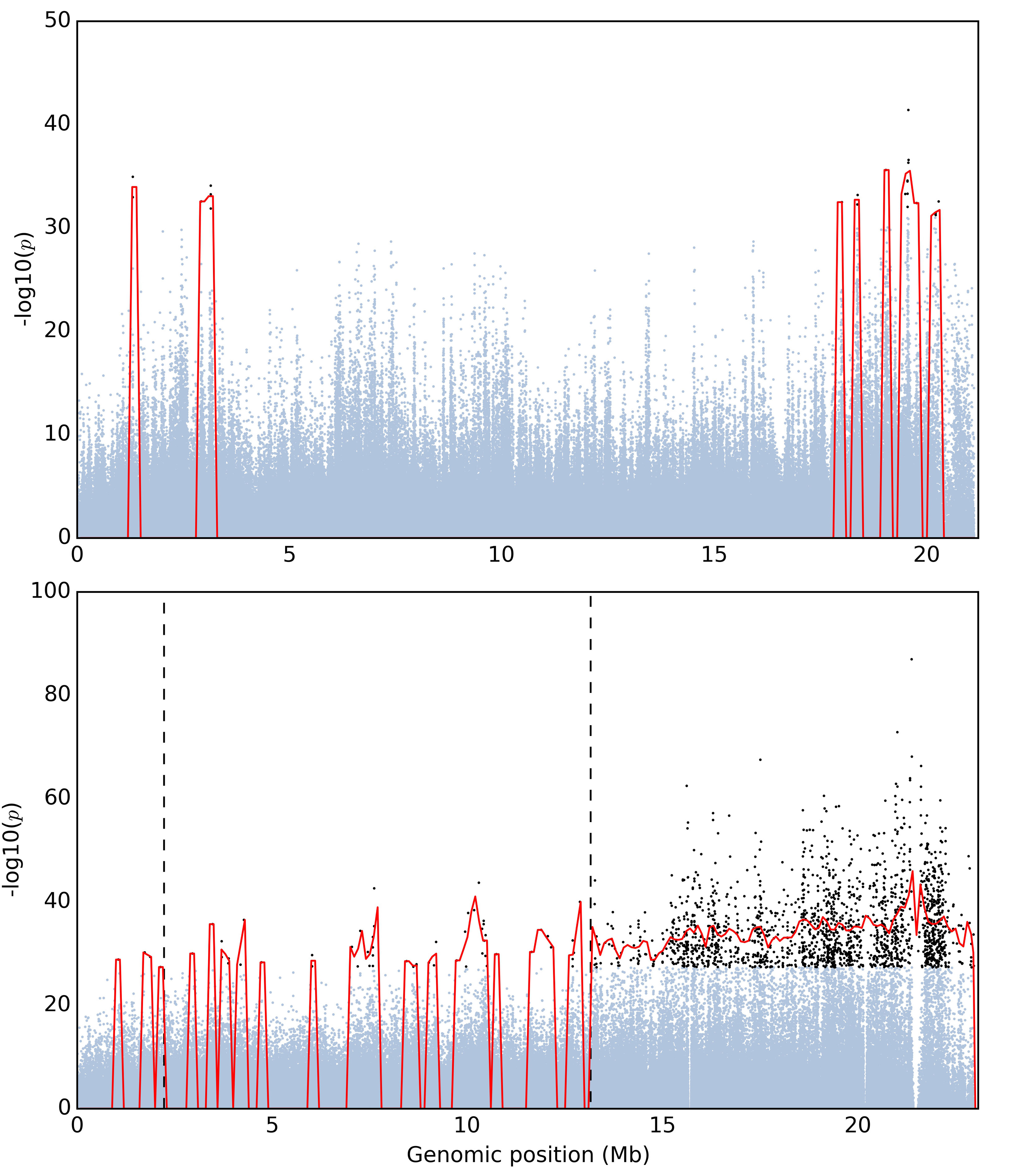
**

**Figure S1** Identification of selected regions in chromosome arm 2L of *D. simulans* (top panel) and *D. melanogaster* (bottom panel). The CMH *p*-values of candidate SNPs (black dots) were averaged across 200kb windows, over sliding intervals every 100kb. Adjacent windows with the average *p*-values above CMH cutoffs (see Materials and Methods) were merged (red lines). Boundaries of the inversion *In(2L)t* is shown in dashed line.

**
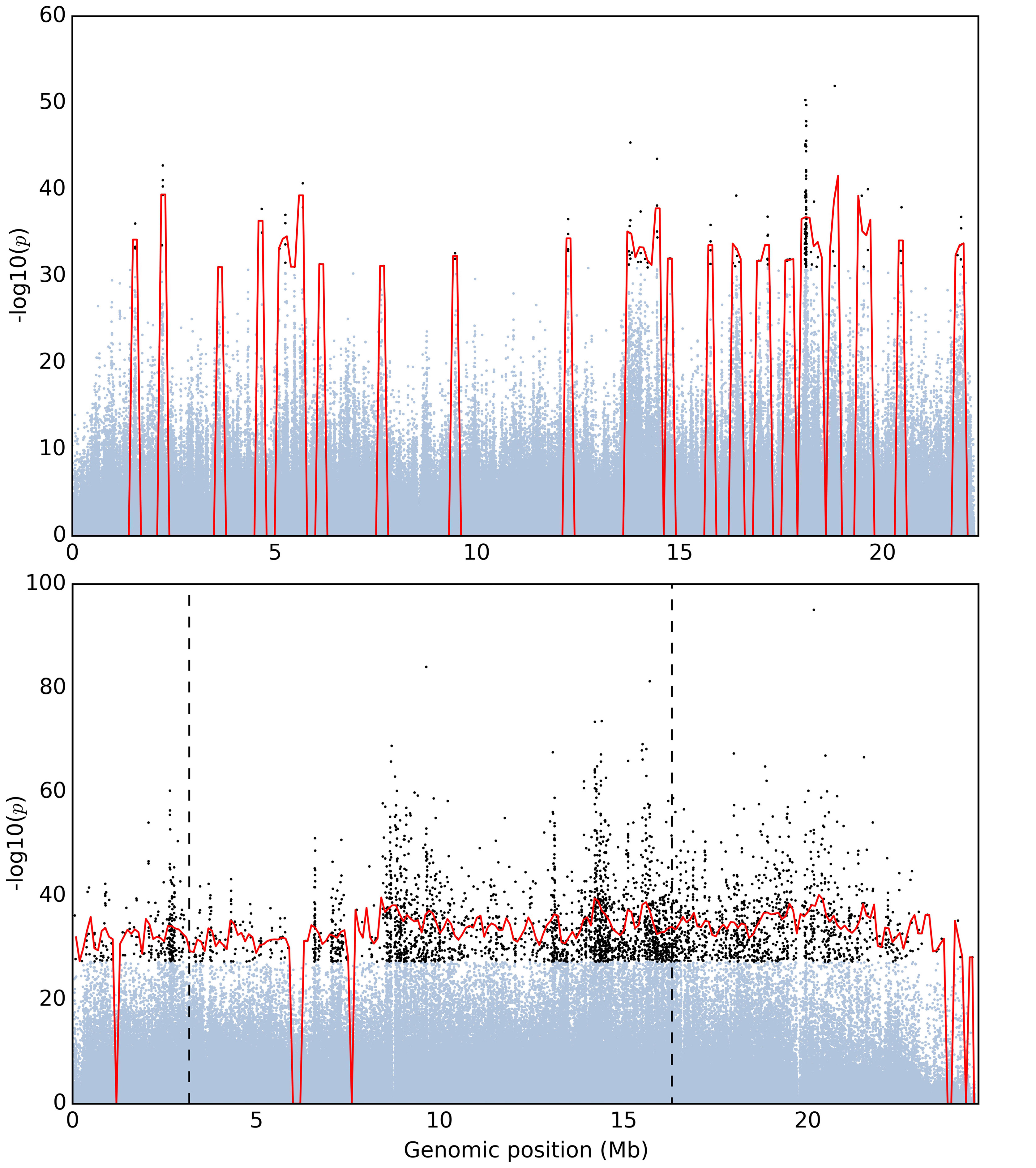
**

**Figure S2** Identification of selected regions in chromosome arm 3L of *D. simulans* (top panel) and *D. melanogaster* (bottom panel). Plot descriptions correspond to Fig. S1. Boundaries of the inversion *In(3L)P* is shown in dashed line.


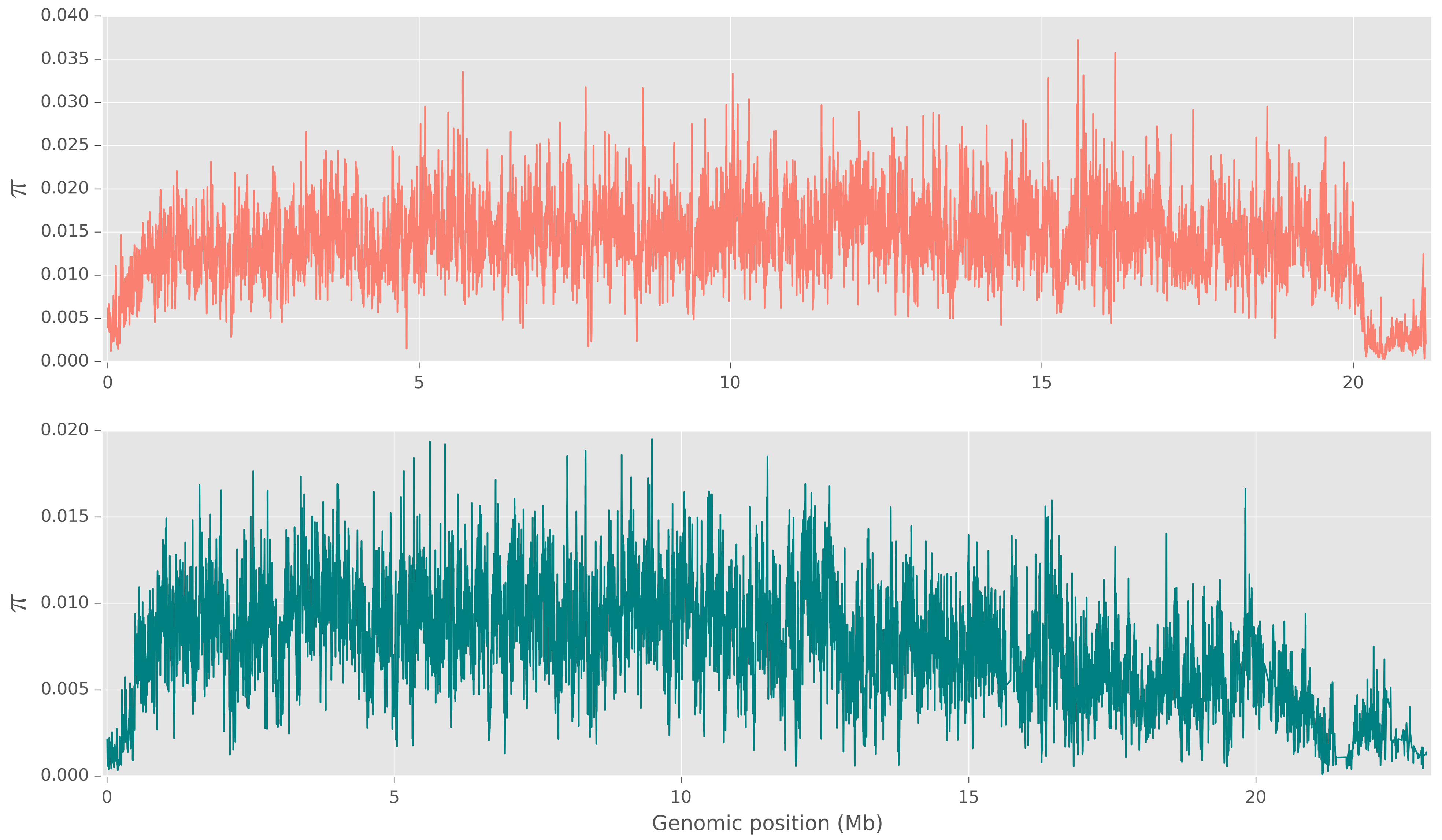


**Figure S3** Nucleotide diversity (π) along the 2L chromosome in the African *D. simulans* (upper panel) and *D. melanoghaster* (lower panel). Analysis of π was performed in Nolte et al. (2013) using PoPoolation (Kofler et al. 2011). Parameters used for π estimation in *D. simulans*: min-count=2, min-coverage=4, max-coverage=120, window size=5000, step size=1000, and in *D. melanogaster*: min-count=3,

min-coverage=6, max-coverage=250, window size=5000, step size=1000.

**
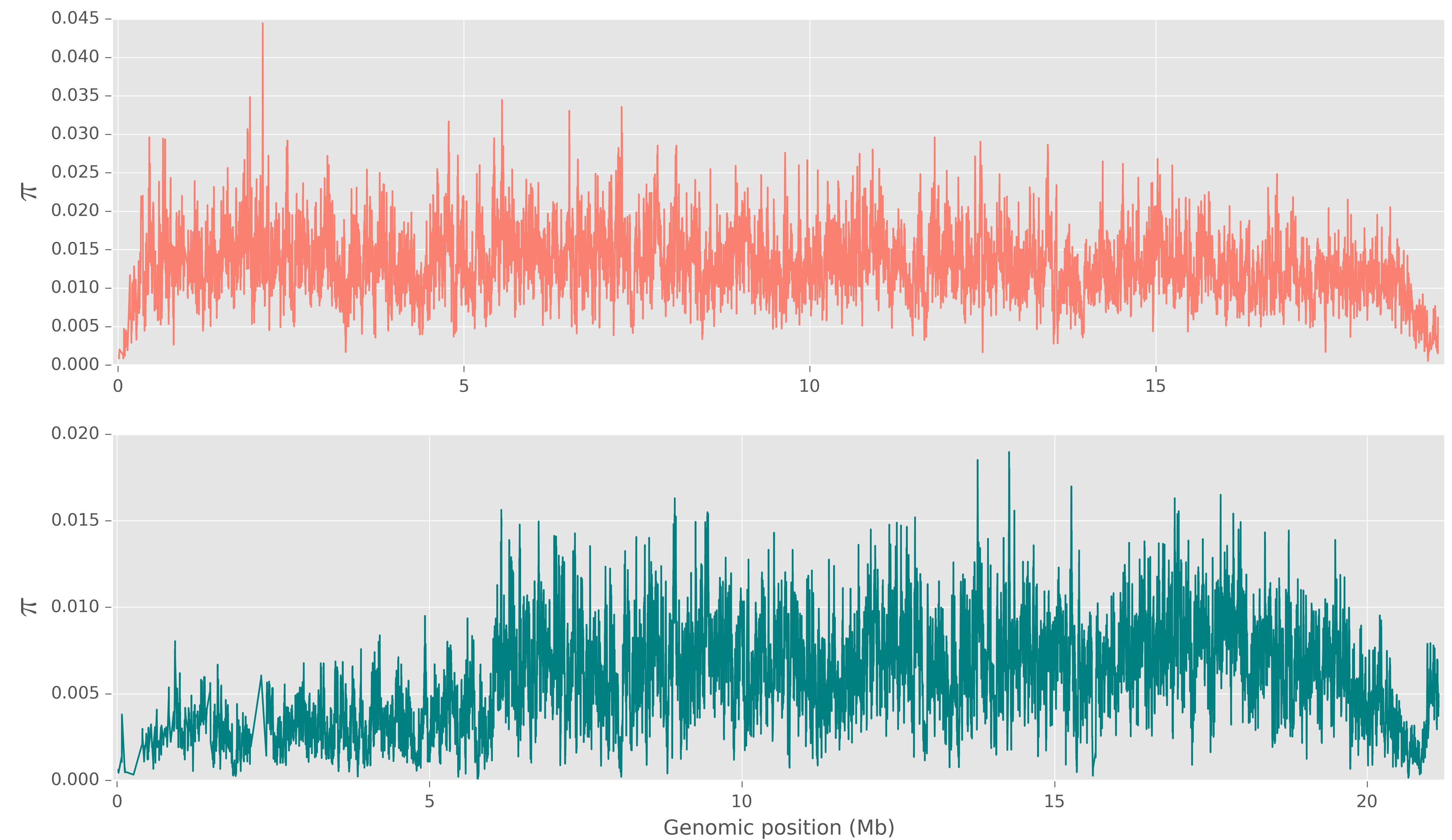
**

**Figure S4** Nucleotide diversity (π) along the 2R chromosome in the African *D. simulans* (upper panel) and *D. melanoghaster* (lower panel). Plot descriptions correspond to Fig. S3.


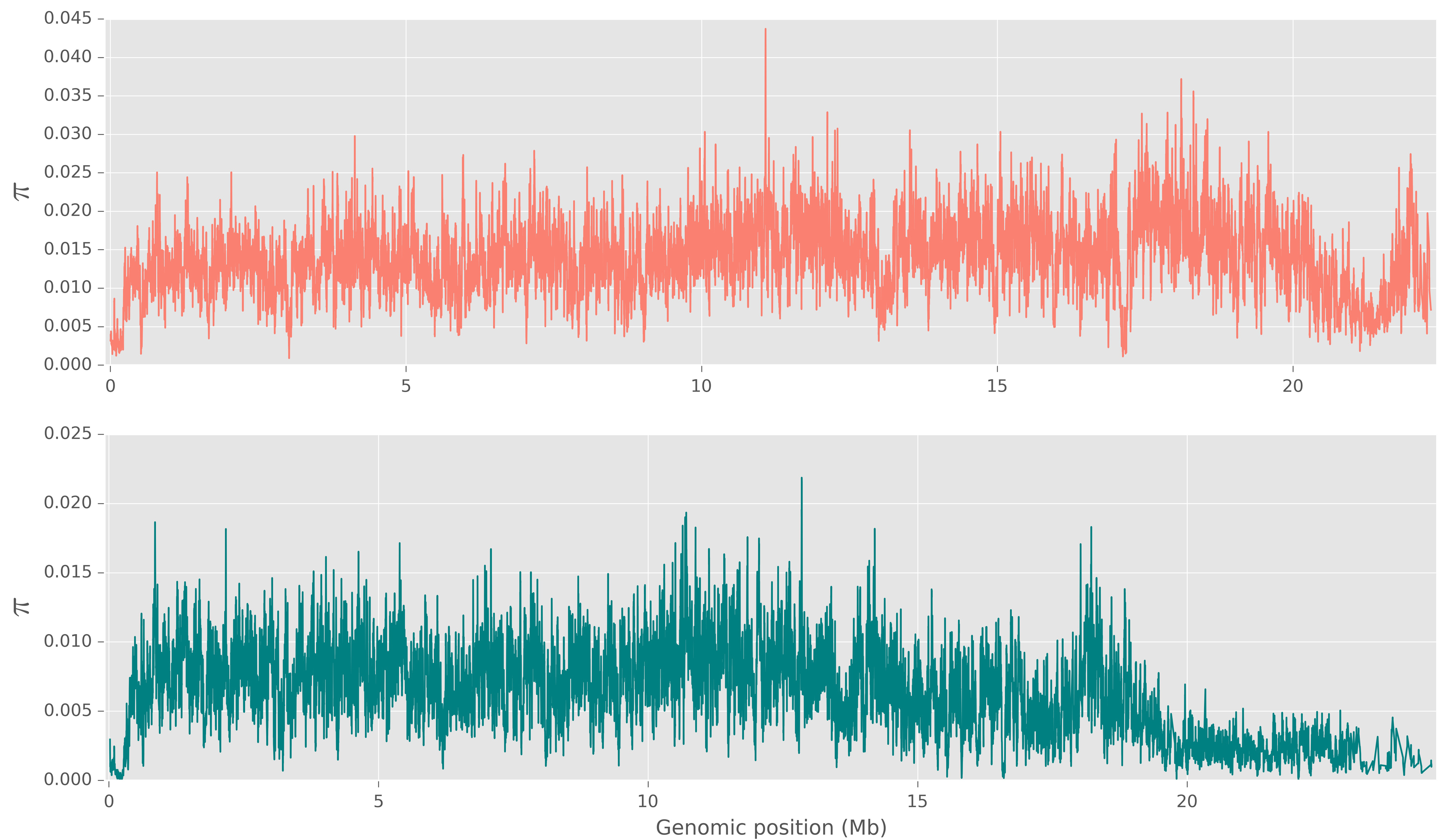


**Figure S5** Nucleotide diversity (π) along the 3L chromosome in the African *D. simulans* (upper panel) and *D. melanoghaster* (lower panel). Plot descriptions correspond to Fig. S3.


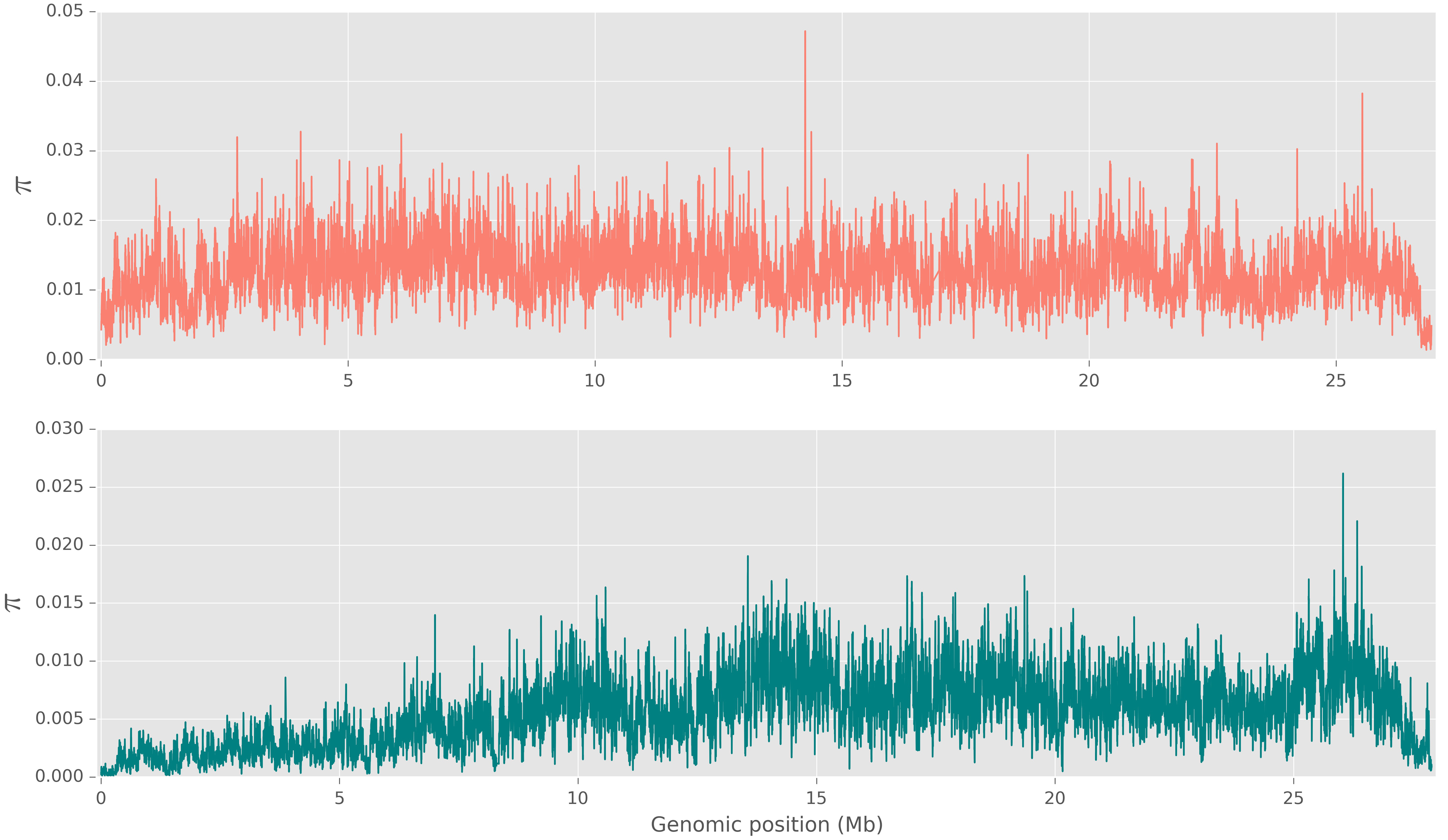


**Figure S6** Nucleotide diversity (π) along the 3R chromosome in the African *D. simulans* (upper panel) and *D. melanoghaster* (lower panel). Plot descriptions correspond to Fig. S3.


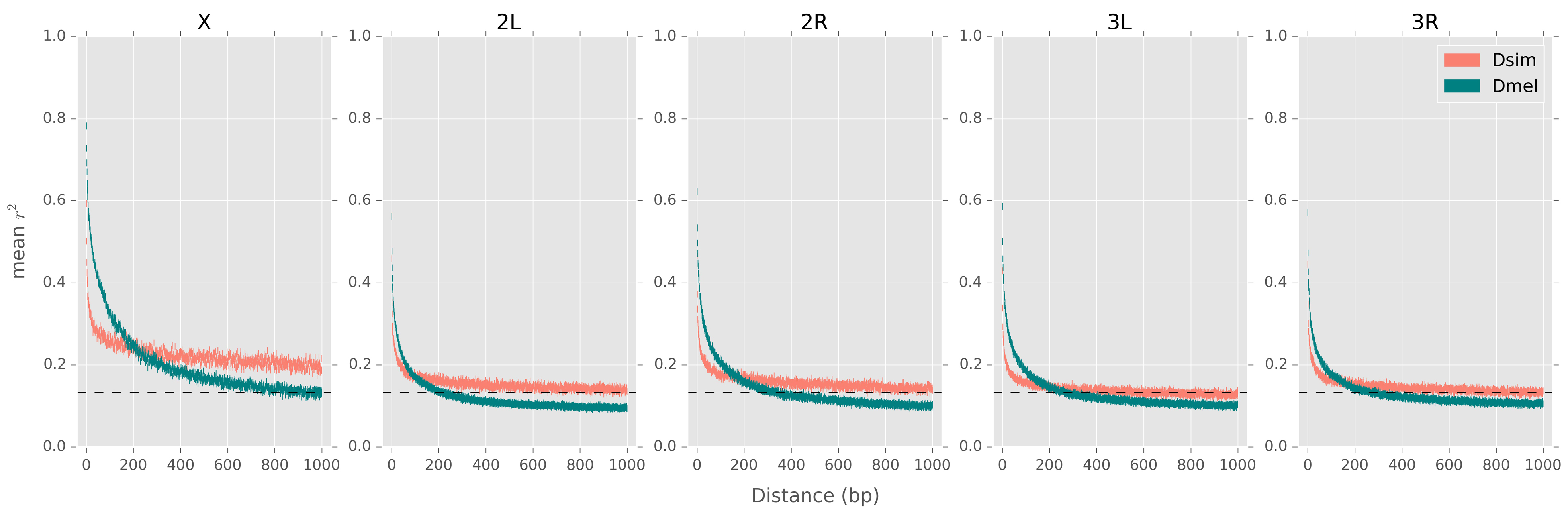


**Figure S7** Linkage disequilibrium decay with physical distance for the major chromosome arms computed for 29 haplotypes of *D. simulans* (Gomez-Sanchez et al. in preparation) and *D. melanogaster* (base haplotypes from Franssen et al. 2015). Linkage (estimated by *r^2^*) was calculated for all possible SNP-pairs for a given physical distance between 0 and 1000 bp. Each SNP should have minimum coverage of 28 for *D. simulans* and 23 for *D. melanogaster*. *r^2^* was estimated using custom script in Franssen et al. 2015. The dotted line specifies the significant threshold (0.132))) which is estimated based on the chi square distribution with 1 degree of freedom and 95% CI (3.841459) divided by the number of haplotypes (29).

**References**

Kofler R, Orozco-terWengel P, De Maio N, Pandey RV, Nolte V, Futschik A, Kosiol C, Schlötterer C. 2011. PoPoolation: A toolbox for population genetic analysis of next generation sequencing data from pooled individuals. PLoS ONE 6: e15925.

Nolte V, Pandey RV, Kofler R, Schlötterer C. 2013. Genome-wide patterns of natural variation reveal strong selective sweeps and ongoing genomic conflict in *Drosophila mauritiana*. Genome Res. 23(1): 99–110.

Franssen SU, Nolte V, Tobler R, Schlötterer C. 2015. Patterns of linkage disequilibrium and long range hitchhiking in evolving experimental *Drosophila melanogaster* populations. Mol Biol Evol. 32:495-509.
